# Abnormal Ventral and Dorsal Attention Network Activity during Single and Dual Target Detection in Schizophrenia

**DOI:** 10.3389/fpsyg.2016.00323

**Published:** 2016-03-08

**Authors:** Amy M. Jimenez, Junghee Lee, Jonathan K. Wynn, Mark S. Cohen, Stephen A. Engel, David C. Glahn, Keith H. Nuechterlein, Eric A. Reavis, Michael F. Green

**Affiliations:** ^1^Desert Pacific MIRECC, VA Greater Los Angeles Healthcare System, Los AngelesCA, USA; ^2^Department of Psychiatry and Biobehavioral Sciences, University of California at Los Angeles, Los AngelesCA, USA; ^3^Department of Psychology, University of Minnesota, MinneapolisMN, USA; ^4^Department of Psychiatry, Yale University, New HavenCT, USA

**Keywords:** schizophrenia, fMRI, visual attention, RSVP, attentional blink

## Abstract

Early visual perception and attention are impaired in schizophrenia, and these deficits can be observed on target detection tasks. These tasks activate distinct ventral and dorsal brain networks which support stimulus-driven and goal-directed attention, respectively. We used single and dual target rapid serial visual presentation (RSVP) tasks during fMRI with an ROI approach to examine regions within these networks associated with target detection and the attentional blink (AB) in 21 schizophrenia outpatients and 25 healthy controls. In both tasks, letters were targets and numbers were distractors. For the dual target task, the second target (T2) was presented at three different lags after the first target (T1) (lag1 = 100 ms, lag3 = 300 ms, lag7 = 700ms). For both single and dual target tasks, patients identified fewer targets than controls. For the dual target task, both groups showed the expected AB effect with poorer performance at lag 3 than at lags 1 or 7, and there was no group by lag interaction. During the single target task, patients showed abnormally increased deactivation of the temporo-parietal junction (TPJ), a key region of the ventral network. When attention demands were increased during the dual target task, patients showed overactivation of the posterior intraparietal cortex, a key dorsal network region, along with failure to deactivate TPJ. Results suggest inefficient and faulty suppression of salience-oriented processing regions, resulting in increased sensitivity to stimuli in general, and difficulty distinguishing targets from non-targets.

## Introduction

Individuals with schizophrenia consistently demonstrate impaired sensory processing, including deficits in early visual perception (Green et al., 1994; Butler et al., 2001) and attention (Nuechterlein et al., 2006; Luck and Gold, 2008). These deficits have clinical consequences in that they are associated with poor functional outcomes (Green et al., 2000; Sergi et al., 2006; Rassovsky et al., 2011). To perceive, identify, and report even a simple visual stimulus (e.g., to read the letter ‘A’) requires processing throughout an extensive network of brain areas involved in perception, cognition, and action. The processing capacity of these systems is limited, so attentional mechanisms exist to select relevant sensory input for processing and to filter out irrelevant input.

Rapid Serial Visual Presentation (RSVP) tasks expose limitations of these attentional mechanisms. In an RSVP task, a series of similar items (e.g., letters) are displayed rapidly in the same spatial location (Reeves and Sperling, 1986; Shapiro, 1994; Chun and Potter, 1995). Viewers can be asked to watch the stream of stimuli for one or more targets (e.g., particular letters) and report them when they appear. When there are two targets (T1 and T2), correct identification of T1 leads to a reduced ability to identify T2 when it appears 200–500 ms later. This effect is known as the attentional blink (AB) (Raymond et al., 1992; Dux and Marois, 2009). The AB is thought to result from a refractory period in which cognitive resources required for the identification of T2 are temporarily unavailable after successful identification of T1. Simple target detection tasks commonly used in studies of schizophrenia, such as oddball detection, are not expected to place as much demand on cognitive resources as do RSVP tasks.

In healthy participants, target detection tasks, including the RSVP, activate two distinct neural networks involved in the allocation of attention: one ventral and one dorsal. The ventral network has been associated with stimulus-driven aspects of attention, including reorienting attention to unexpected or salient (e.g., target) stimuli. Regions of the ventral network include anterior insula (AI), anterior cingulate cortex (ACC), and temporo-parietal junction (TPJ) (Kim, 2014; Vossel et al., 2014). The dorsal network is associated with voluntary, sustained orienting of attention. Regions of the dorsal network include lateral frontal cortex (LFC) as well as anterior and posterior intraparietal cortex (aIPC and pIPC, respectively) (Marois et al., 2000, 2004; Marcantoni et al., 2003; Kranczioch et al., 2005; Johnston et al., 2012; Jiang et al., 2013).

Direct investigations of attentional processing using RSVP tasks in schizophrenia are scarce, with only a handful of behavioral studies (Cheung et al., 2002; Li et al., 2002; Wynn et al., 2006; Mathis et al., 2011) and one electrophysiological study (Mathis et al., 2012) published to date. While areas of both the ventral and dorsal attention networks are involved in attentional processing during RSVP tasks, it is possible that only one or a few of the areas within those networks functions abnormally in schizophrenia during RSVP, leading to the previously reported behavioral deficits. No study to date has investigated RSVP-related processing in schizophrenia with fMRI or any other technique capable of determining *where* in the brain processing is abnormal.

In simpler target detection tasks (e.g., single target and oddball detection), patients with schizophrenia show disruptions in both the ventral and dorsal networks (e.g., Gur et al., 2007; Hasenkamp et al., 2011). However, these simpler target-detection tasks do not push attentional systems to their limits in the way that demanding RSVP tasks do, so it is possible that previous studies have not delineated which attentional brain areas are dysfunctional in schizophrenia.

Recent studies in schizophrenia have highlighted dysfunction in the ventral network in particular (e.g., White et al., 2013; Wynn et al., 2015), rather than in the dorsal network. The ventral network is closely related to the so-called “salience network.” The salience network has been implicated in the pathophysiology of schizophrenia, with dysfunction resulting in the incorrect assigning of salience which can in turn lead to the key symptoms of schizophrenia, including delusions (Palaniyappan and Liddle, 2012). Tasks such as the RSVP can potentially be useful for parsing out the relative contributions of the ventral (salience) and dorsal networks to attentional deficits seen in schizophrenia, and how deficits in those networks relate to the clinical features of the disease.

The aim of the current study was to use fMRI to identify the differential roles of the ventral and dorsal attentional networks on single target and dual target RSVP tasks in patients with schizophrenia. Based on prior work using the RSVP task in healthy controls and other target detection tasks in schizophrenia, we hypothesized that patients would exhibit abnormally increased regional activation in the ventral attention system (specifically AI, ACC, and TPJ) and decreased activity in the dorsal system (LFC, aIPC, and pIPC) during RSVP tasks. We used a region of interest (ROI)-based analytical approach to focus on the activity in these specific areas during RSVP task performance to test this hypothesis.

## Materials and Methods

### Participants

Thirty patients with schizophrenia (7 female) and 29 normal controls (6 female) were recruited for the study. All participants were between 18 and 60 years of age. All participants had normal or corrected-to-normal vision as assessed using the mini-Snellen eye chart. Schizophrenia patients were recruited from outpatient treatment clinics at the VA Greater Los Angeles Healthcare System (GLA) and through presentations in the local community. Normal control participants were recruited through internet postings. All participants were administered the Structured Clinical Interview for DSM-IV Axis I Disorders (SCID) (First et al., 1997b). Eligibility for inclusion as a patient was a diagnosis of schizophrenia. Exclusion criteria for all participants included substance abuse or dependence in the last six months, IQ < 70 based on review of medical records, a history of loss of consciousness for more than one hour, an identifiable neurological disorder, and not sufficiently fluent in English to demonstrate understanding of study procedures. Additional exclusion criteria for potential normal controls included a first-degree relative with schizophrenia or another psychotic disorder, a personal history of schizophrenia or other psychotic disorder, bipolar disorder, or recurrent depression, or diagnosis of avoidant, paranoid, schizoid, or schizotypal Axis II disorders based on the Structured Clinical Interview for DSM-IV Axis II Disorders (SCID-II) (First et al., 1997a). Clinical symptoms were assessed in patients using the 24-item University of California, Los Angeles, CA, USA (UCLA) version of the Brief Psychiatric Rating Scale (BPRS) (Ventura et al., 1995) and Scale for the Assessment of Negative Symptoms (SANS) (Andreasen, 1982). For the BPRS we report total scores and means for the “positive symptom” and “negative symptom” factors (Kopelowicz et al., 2008). For the SANS we report four global scales (not including attention; Blanchard and Cohen, 2006): Affective Flattening, Alogia, Avolition-Apathy, and Anhedonia-Asociality.

All clinical interviewers were trained through the Treatment Unit of the Department of Veterans Affairs VISN 22 Mental Illness Research, Education, and Clinical Center (MIRECC) to a minimum kappa of 0.75 for key psychotic and mood items. The study protocol was reviewed and approved by the institutional review boards of the GLA and the UCLA. All participants had the capacity to give informed consent (e.g., not under county conservatorship) and provided written informed consent after all procedures were fully explained and understanding of procedures was demonstrated through completion of an informed consent comprehension assessment tool.

### Experimental Design

**FIGURE 1:**
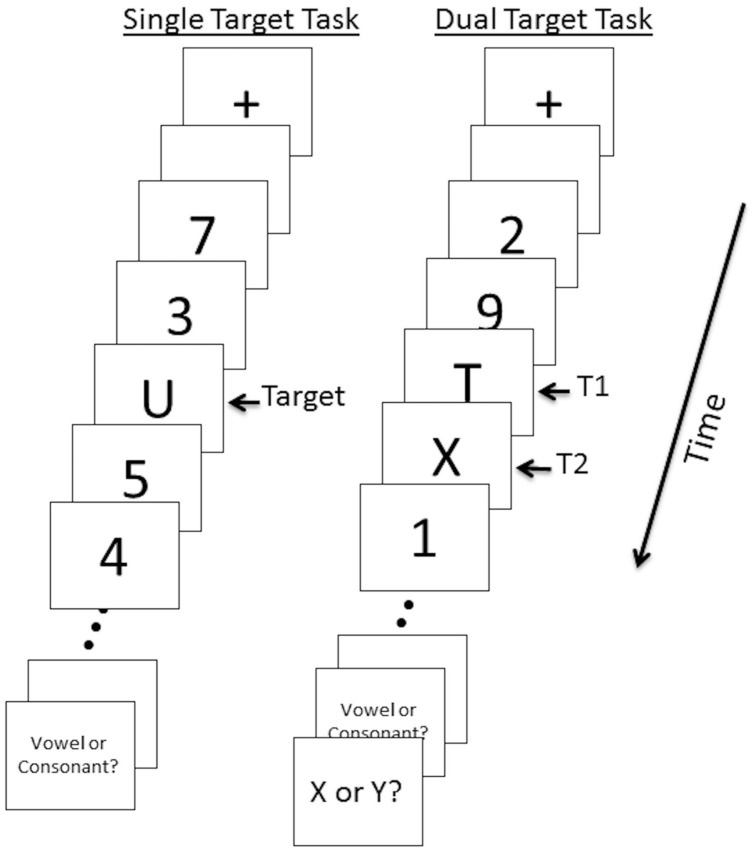
**Single and dual target RSVP task illustration.** Twenty stimuli (targets and distractors) per trial were presented for 85 ms with a 15 ms interstimulus interval. A 5000 ms response period prompted participants to indicate whether T1 was a vowel or a consonant (single and dual target tasks) and whether T2 was X or Y (dual target task). In the dual target task trial shown here, T2 is presented at lag 1.

Participants performed two RSVP tasks given in a set order during event-related fMRI: a *single target detection* task followed by a *dual target detection* task (**Figure 1**). In the single target task, twenty stimuli (including targets) were presented at the center of the screen in rapid sequence. Total duration of each trial was 2000 ms (∼85 ms per stimulus with ∼15 ms interstimulus interval). Each trial began with a fixation cross displayed in the center of the screen for 300 ms followed by two 100 ms fixation point flashes, followed by a 300 ms blank screen. Letters (uppercase letters A, C, E, J, O, R, T, or U) served as targets and numbers (1–9) served as distractors. All stimuli were black, presented on a white background. After the RSVP sequence, subjects were asked whether the target letter was a Vowel or Consonant. Subjects responded using their right hand by pressing one of two corresponding buttons. The response period was 2500 ms and was followed by a 2500 ms fixation period. A total of 34 trials were administered. Eight “null” trials consisting of a central fixation point displayed for 8000ms were also included. Null trials contributed to the implicit baseline. A practice session of 8 trials was conducted prior to the task.

The dual target task followed identical procedures as the single target task with the following exceptions. The dual target task included a second target letter (X or Y) with 0, 2, or 6 distractors between the first and second target (T1 and T2). We refer to these as lag 1, lag 3, and lag 7, respectively. For lag 1, T2 occurred 100 ms after T1; for lag 3, T2 occurred 300 ms after T1; for lag 7, T2 occurred 700 ms after T1. Thus, the AB was expected to be most prominent in lag 3 trials. During the response period participants were asked whether the first target was a Vowel or Consonant (2500 ms duration) and whether the second target was X or Y (2500 ms duration). The total response period was 5000 ms. Thirty four trials of each lag and 34 null trials were presented in pseudo-randomized order (Dale and Buckner, 1997) across four runs; each run consisted of 34 trials.

All stimuli were developed and presented using E-Prime 1.1 software (Psychological Software Tools, Pittsburgh, PA, USA) installed on a PC. All tasks were presented with MR-compatible LCD goggles (Resonance Technology, Northridge, CA, USA). Corrective lenses were applied if necessary.

### fMRI Data Acquisition

Imaging was performed on a Siemens (Erlangen, Germany) 3-T Trim Trio scanner located at the UCLA Ahmanson-Lovelace Brain Mapping Center. A T2^∗^-weighted blood oxygen level-dependent (BOLD) gradient echo planar imaging (EPI) sequence was obtained for each activation task run (TR = 2000 ms; TE = 30 ms; flip angle = 75°; 33 contiguous AC–PC aligned slices; slice thickness 4mm; matrix 64 × 64; FOV 220 mm). For anatomical reference, two sets of structural images were acquired: a T1 weighted magnetization prepared rapid-acquisition gradient echo (MPRAGE) image [TR = 1900 ms; TE = 3.43 ms; flip angle = 9°; 160 sagittal slices; slice thickness 1 mm; matrix 256 × 256; FOV 256 mm]; and a T2-weighted matched-bandwidth high-resolution scan with the same slice prescription as the EPI [TR = 6540 ms; TE = 13 ms; flip angle = 120°; 33 axial slices; slice thickness 4 mm; matrix 128 × 128; FOV 220 mm].

The fMRI data were pre-processed and analyzed using the FMRIB Software Library (FSL v5.0; Analysis Group, Oxford, UK). Data were spatially smoothed using an 8mm full-width at half-maximum Gaussian kernel and temporally filtered using a 100 s cut-off highpass filter. Images were skull stripped using BET (Smith, 2002). Movement parameters, calculated using MCFLIRT (Jenkinson and Smith, 2001), were modeled as nuisance covariates. Translational movement parameters of the final sample did not exceed 2 mm. Using FLIRT (FMRIB’s Linear Image Registration Tool v6.0) (Jenkinson and Smith, 2001), functional images were registered first to the co-planar matched-bandwidth high resolution T2-weighted image, then to the T1-weighted MPRAGE via 6-parameter rigid-body transformation, and finally to standard Montreal Neurological Institute (MNI) space using a 12-parameter affine transformation. Individual subjects with missing MPRAGE images (two patients, four controls) were registered to a group-specific common brain in the intermediate step. The group-specific common brains were generated via an iterative averaging processing using FLIRT and the fslmaths tool.

### Behavioral Data Analysis

Statistical analyses of behavioral data were conducted using SPSS (Armonk, NY, USA: IBM Corporation). The key dependent variable for the single target task was the proportion of targets detected correctly, and groups were compared using an independent-samples *t*-test. The key dependent variable for the dual target task was conditional probability at each lag *p*(T2| T1), which is commonly used for AB tasks. This value is the probability of correctly detecting the second target (T2) given the correct detection of the first target (T1). Data from this task were analyzed using a repeated measures analysis of variance (rmANOVA), with lag as the within subjects factor and group as the between subjects factor. Significant effects were further evaluated with *post hoc* comparisons.

### fMRI Data Analysis

Analysis of functional imaging data was performed using a multi-stage general linear model approach with FEAT (FMRI Expert Analysis Tool v6.0) and a timing model based on a double-gamma hemodynamic response function (HRF). In the individual first-level analyses, event modeling was performed for the single target task and each dual target task run separately. For the single target task, all trials in which participants correctly detected the target were included in the analysis. Incorrect trials and the response period following each RSVP sequence were modeled as nuisance variables. Linear contrasts of correct trials versus the implicit baseline were created for each subject. For the dual target task, each lag (lag 1, lag3, lag7) was modeled as an explanatory variable. All trials in which participants correctly detected the first target (T1) were included, regardless of correct detection of the second target (T2), because correct T1 detection is needed for an AB to occur. Incorrect T1 trials and the response period were modeled as nuisance variables. A linear contrast of all trials across lags combined versus the implicit baseline was conducted to examine the overall effect of dual target detection. Linear contrasts for each lag versus baseline, as well as lag versus lag contrasts, were also conducted to isolate effects by lag. The four runs for each participant were then averaged together in a higher-level fixed effects model. After averaging, the single and dual target task contrasts were included in an additional higher level model. Linear contrasts were created for the dual target task versus the single target task, to examine the effect of adding a second target to the RSVP sequence.

**FIGURE 2:**
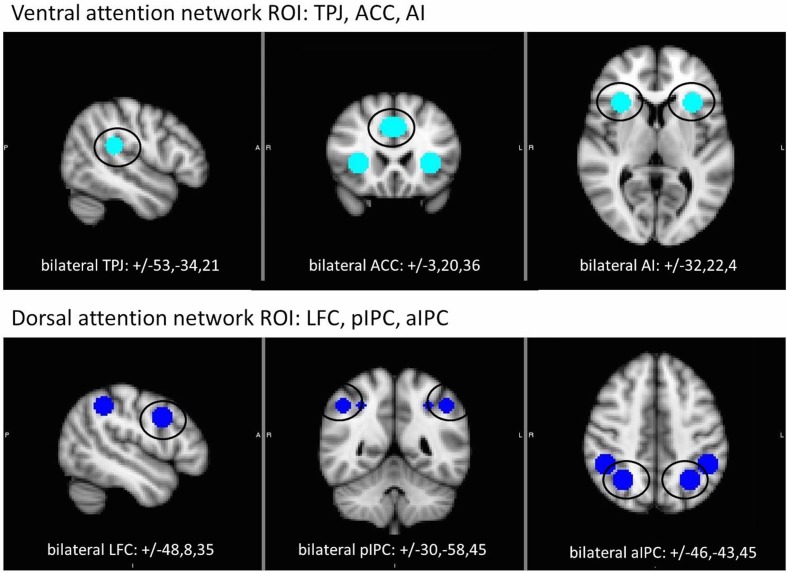
**Location of regions of interest (ROI) within the ventral (top) and dorsal (bottom) attention networks.** Each ROI (9 mm sphere) centered on coordinates shown in Montreal Neurological Institute (MNI) standard space. ACC, anterior cingulate cortex; AI, anterior insula; aIPC anterior intraparietal cortex; LFC, lateral frontal cortex; pIPC, posterior intraparietal cortex; TPJ, temporo-parietal junction.

We used an ROI approach to focus analysis on contributions of ventral and dorsal attention network regions during single and dual target detection. Spherical ROIs (9 mm diameter) were created around peak coordinates identified in prior fMRI studies (Marois et al., 2004; Kim, 2014) to create bilateral ROIs for ACC, AI, and TPJ (ventral network) and LFC, aIPC, and pIPC (dorsal network). All ROIs and coordinates are displayed in **Figure 2**. For each task, mean beta values were extracted from each ROI and analyzed using a group X ROI rmANOVA with accuracy included as a covariate. Separate analyses were conducted for each attention network. For analysis of the dual target task by lag, a group X lag (lag 1, lag 3, lag 7) rmANOVA was performed for each ROI. Parallel analysis with the dual target versus single target task contrasts was performed to more closely examine the specific effect of dual target processing over and above single target. To further assess the AB, lag 3 (i.e., maximal AB) was contrasted with lags 1 and 7 in 2 (group) × 3 (ROI) rmANOVAs for each network.

## Results

Technical problems with behavioral or image acquisition resulted in missing runs for six patients and three controls, and we did not include subjects who were missing any runs. In addition, two patients and one control had excessive movement artifacts (translational > 2 mm) and one patient had poor behavioral performance (defined as dual target task accuracy less than two standard deviations below the group mean). Following these exclusions, 21 patients with schizophrenia (four female) and 25 normal controls (five female) were included in analyses.

Sample demographic and clinical data are shown in **Table 1**. There were no differences between groups in terms of age, sex, ethnicity, or personal and parental years of education. Because most of the patient participants were recruited from VA clinics, the sample is predominantly male. Patients were clinically stable outpatients. Twenty patients were receiving atypical antipsychotic medication, with no changes in dosage or type within the previous 6 weeks. One patient was not receiving any antipsychotic medication at the time of testing. The mean daily dose (in chlorpromazine equivalents) is shown in **Table 1**.

**Table 1:** Sample demographic and clinical characteristics.

Characteristic	Patient *n* = 21	Control *n* = 25	Statistic	Df	*P*-value
	**Mean (*SD*)**	**Mean (*SD*)**			
Age	42.2 (10.8)	41.4 (7.6)	*t* = 0.28	44	0.78
Years of education	13.5 (1.6)	14.2 (1.9)	*t* = –1.36	44	0.18
Average parental education^∗^	13.6 (3.0)	13.0 (1.9)	*t* = 0.71	39	0.49
	**No. (%)**	**No. (%)**			
Gender (male/female)	17/4	20/5	*X*^2^ = 0.01	1	0.94
Ethnicity			*X*^2^ = 0.52	2	0.78
Caucasian	13 (62%)	13 (52%)			
African American	7 (33%)	10 (40%)			
Other	1 (5%)	2 (8%)			
	**Mean (SD)**				
Medication dosage (chlorpromazine equivalent in mg/day)	337.65 (240.11)				
BPRS					
Total score	46.52 (10.8)				
Positive symptoms	2.46 (0.8)				
Negative symptoms	1.73 (0.9)				
SANS					
Affective flattening	1.57 (1.5)				
Alogia	0.52 (1.0)				
Avolition	2.52 (1.2)				
Anhedonia	2.62 (1.2)				


*^∗^Data available for 19 patients, 22 controls. SD, standard deviation; No., number; mg, milligrams; BPRS, Brief Psychiatric Rating Scale; SANS, Scale for the Assessment of Negative Symptoms.*

### Behavioral Data

**FIGURE 3:**
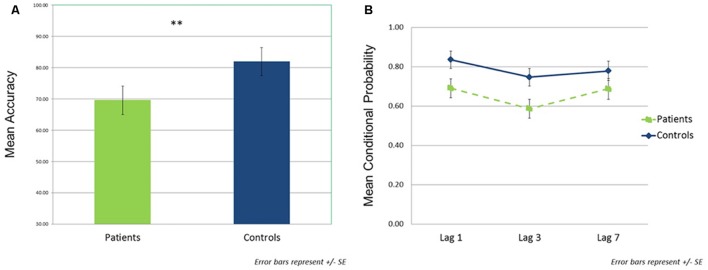
**Behavioral performance data.** **(A)** Single target task performance (mean accuracy) by group. **(B)** Dual target task performance by group as a function of lag. Assessed using the probability of correct T2 identification given correct T1 identification (conditional probability). ^∗∗^*p* < 0.01.

In the single target task, patients detected a significantly lower percentage of targets than controls (patients: *M* = 68.1, *SD* = 21.9; controls: *M* = 85.1, *SD* = 16.4; *t*(44) = –3.00, *p* < 0.01, *d* = 0.87; **Figure 3A**). For the dual target task, conditional probability data across lags are shown in **Figure 3B**. The rmANOVA revealed significant main effects of group [*F*(1,44) = 4.34, *p* < 0.05, $\eta_{p}^{2}$ = 0.09] and lag [*F*(2,88) = 12.14, *p* < 0.01, $\eta_{p}^{2}$ = 0.22], but no significant group × lag interaction [*F*(2,88) = 1.65, *p* = NS, $\eta_{p}^{2}$ = 0.04]. Across lags, patients showed lower conditional probabilities relative to controls [*t*(44) = –2.08, *p* < 0.05, *d* = 0.63]. In addition, the combined groups demonstrated reduced accuracy at lag 3 compared to either lag 1 [*t*(45) = –5.27, *p* < 0.001, *d* = 0.78] or lag 7 [*t*(45) = –2.96, *p* < 0.005, *d* = 0.44]; lags 1 and 7 did not differ from each other [*t*(45) = 1.58, *p* = 0.12, *d* = 0.23].

### fMRI Data

Results of all ROI analyses are provided in Supplementary Materials. Findings are summarized below.

#### Single Target Task

**FIGURE 4:**
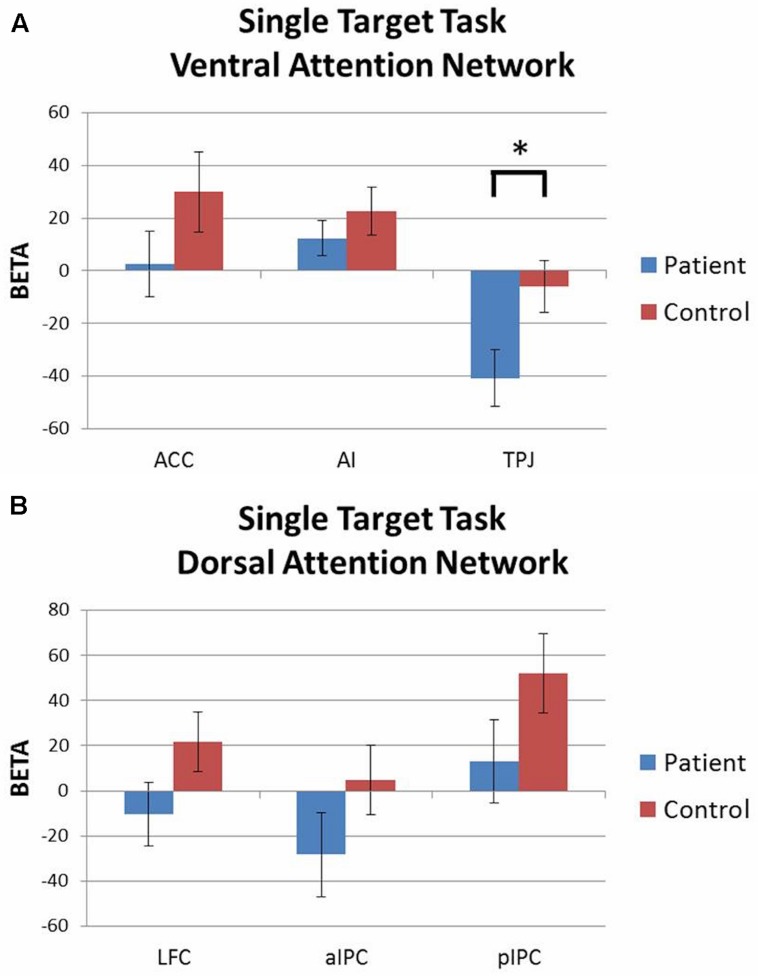
**Single target task ROI analysis by network (contrast: single target task > baseline).** **(A)** Ventral attention network: patients showed significantly greater deactivation of TPJ than controls. **(B)** Dorsal attention network: no significant group differences. ^∗^*p* < 0.05.

Activation patterns were evaluated with 2 (group) × 3 (ROI) rmANOVAs for each attention network with accuracy included as a covariate. For the ventral network there was a significant group × ROI interaction [*F*(2,86) = 3.05, *p* = 0.05, $\eta_{p}^{2}$ = 0.07] driven by greater deactivation of TPJ in patients than controls. Both groups had similar levels of activation in ACC and AI (**Figure 4A**). For the dorsal network, both groups showed similar levels of activation in LFC, aIPC, and pIPC (**Figure 4B**). There were no significant main effects of group or ROI, and no group × ROI interaction effects.

#### Dual Target Task

**FIGURE 5:**
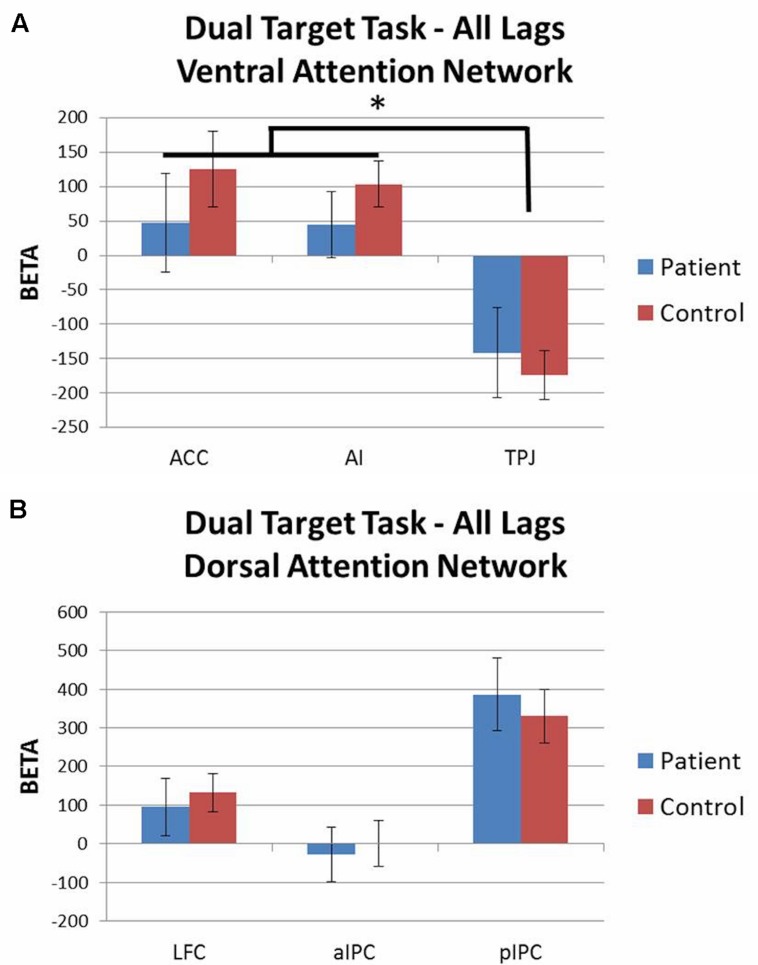
**Dual target task ROI analysis by network (all lags combined; contrast: dual target task > baseline).** **(A)** Ventral attention network: both groups showed activation of ACC and AI, but deactivation of TPJ. **(B)** Dorsal attention network: no significant group differences. ^∗^*p* < 0.05.

Activation patterns for all lags combined were evaluated with 2 (group) × 3 (ROI) rmANOVAs for each attention network including accuracy as a covariate. For both the ventral and dorsal networks there were no significant main effects of group and no group × ROI interaction effects. For the ventral network there was a main effect of ROI [*F*(2,86) = 5.82, *p* < 0.01, $\eta_{p}^{2}$ = 0.12] as both groups showed similar levels of activation in ACC and AI, but deactivation of TPJ (**Figure 5A**). For the dorsal network, both groups showed similar levels of activation in LFC, aIPC, and pIPC (**Figure 5B**). These results did not significantly change when the dual target task (all lags combined) was contrasted against the single target task.

**FIGURE 6:**
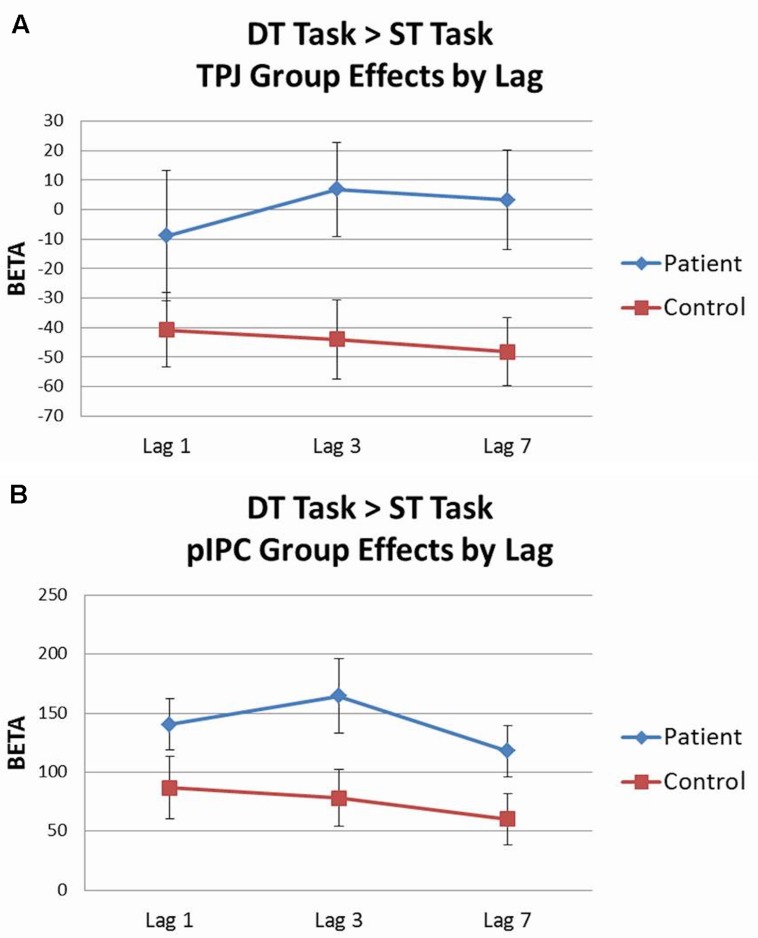
**Dual target task vs. single target task ROI analysis by lag.** **(A)** Controls showed significantly greater deactivation of temporo-parietal junction (TPJ) than patients across lags. **(B)** Patients showed greater activation of posterior intraparietal cortex (pIPC) than controls across lags.

Analysis of the dual target task by lag was conducted using 2 (group) × 3 (lag 1, lag 3, lag 7) rmANOVAs for each ROI with accuracy included as a covariate. Activation patterns for each lag were largely equivalent to those seen from the all lags combined analyses. When the dual target task lags were contrasted against the single target task in a parallel 2 × 3 rmANOVA, significant effects were observed for two regions. Within the ventral network, there was a main effect of group for TPJ [*F*(1,43) = 6.42, *p* < 0.05, $\eta_{p}^{2}$ = 0.13], with controls showing deactivation of TPJ across lags, whereas patients did not (see **Figure 6A**). Within the dorsal network, there was a main effect of group for pIPC [*F*(1,43) = 4.20, *p* < 0.05, $\eta_{p}^{2}$ = 0.09], with patients showing greater activation of pIPC than controls across lags (see **Figure 6B**).

To further assess the AB, lag 3 (i.e., maximal AB) was contrasted with lags 1 and 7 in 2 (group) × 3 (ROI) rmANOVAs for each network including accuracy as a covariate. There were no significant main effects of group or ROI, and no group × ROI interaction effects, for either network.

### Correlations with Clinical Data

In *post hoc* analyses we explored the relationship between medication dose equivalents and clinical symptom ratings with ROI activity within regions of the ventral and dorsal attention network as well as task performance. We focused on regions that were significantly different between groups, namely, TPJ for the single target task, and TPJ and pIPC for the dual target versus single target task. Non-parametric analysis (Spearman correlations) was chosen to minimize potential effects of data outliers and of a non-Gaussian distribution of the data. We found no significant correlations between medication dosage (chlorpromazine equivalents) or either symptom scale (BPRS total or factor scores or SANS global scale scores) with ROI activity or task accuracy that survived correction for multiple comparisons.

## Discussion

We utilized event-related fMRI to identify regional brain activity associated with impaired performance on an RSVP paradigm in schizophrenia in specific regions of the dorsal and ventral attention networks. Across tasks, patients demonstrated poorer performance in target detection than controls, and both groups showed the expected AB effect behaviorally (i.e., poorer dual target performance at lag 3 than either lag 1 or lag 7). Patients exhibited aberrant activation patterns within both the ventral and dorsal networks, depending on the task. Overall, the results did not fully support our initial hypothesis that patients would show abnormally high activity in the ventral attention system (AI, ACC, and TPJ) and abnormally low activity in the dorsal system (LFC, aIPC, and pIPC). Rather, the pattern of results suggests a more complex and nuanced set of differences between patients and controls during RSVP tasks.

During the single target task, patients exhibited significantly greater deactivation in TPJ, a key region of the ventral attention network, even after controlling for accuracy differences in the groups. In contrast, the pattern of activation for patients in the dorsal attention network was similar to controls. Some suggest that the TPJ, when activated, acts as a neural “circuit breaker” to interrupt sustained attention processes supported by the dorsal network so novel relevant stimuli can be processed (Corbetta and Shulman, 2002). Conversely, suppression of the TPJ during target detection tasks may offer a focusing mechanism to maintain goal-directed behavior in the presence of irrelevant distractors. Not surprisingly, activity in TPJ is suppressed during “top–down” visual attention tasks (Todd et al., 2005; Shulman et al., 2007). Therefore, greater deactivation of TPJ in patients during the single target task may reflect more effort or inefficient effort to maintain sustained attention (Harrison et al., 2007).

During the dual target task, overall (i.e., with all lags combined) the pattern of activation in both dorsal and ventral attention networks was similar between patients and controls. Consistent with prior RSVP studies, both groups showed activation of dorsal network regions, especially pIPC, and deactivation of key ventral network regions, especially TPJ (Marcantoni et al., 2003; Shulman et al., 2003, 2007; Marois et al., 2004; Kranczioch et al., 2005). When examining the individual lags of the dual target task, controlling for single target task activation, we found significant group differences in TPJ and pIPC. Patients showed less deactivation of TPJ and greater activation of pIPC than controls across lags.

The pIPC region may be particularly relevant to the modulation of selective attention by top-down biases such as expectations and behavioral goals while maintaining a stable “priority” map of the visual environment to monitor saliency (Corbetta et al., 2008; Ptak, 2012). In line with this view, we observed that patients showed overactivation of pIPC in the dorsal network, along with failure to deactivate TPJ in the ventral network during the dual- vs. single-target task, which could reflect faulty coordination between these two critical regions (Shulman et al., 2007; Corbetta et al., 2008). However, this interpretation remains speculative at this time because these activation differences were not observed when examining dual target alone and the current study did not explicitly test functional connectivity of these networks.

Overall, our results indicate abnormal activity in key regions of the ventral attention network in schizophrenia, particularly TPJ, with the deficit being most prominent in single target processing. These findings contribute to a growing body of literature indicating disrupted salience processing (White et al., 2013; Uddin, 2015; Wynn et al., 2015) and abnormalities of the salience network (Moran et al., 2013; Palaniyappan et al., 2013; Manoliu et al., 2014; see also Baker et al., 2014) in schizophrenia. Aberrant salience processing may be involved in core disease pathophysiology, whereby faulty assignment of salience to internally generated mental events form the basis of positive symptoms such as delusions and hallucinations (Palaniyappan and Liddle, 2012). Downstream consequences of this aberrant salience model would include difficulty sustaining goal-directed attention to behaviorally relevant stimuli in the external world.

Most of the studies cited above reporting abnormalities of the salience network in schizophrenia have utilized resting-state imaging methodologies with findings focused on the anterior insula. Our findings highlighting abnormal activation of TPJ in the context of impaired target detection would thus benefit from replication. In addition, direct investigation into the relative contributions of each of the attention networks to impaired attention processing in schizophrenia, including connectivity within and between the networks, requires further investigation.

The current study had several limitations. Our patient sample was comprised of chronic outpatients who were taking antipsychotic medications. It is not known whether similar patterns of regional activation differences would be observed in recent-onset or unmedicated individuals. In exploratory analyses we examined correlations for ROI activity and behavioral performance with symptom ratings scales and medication dose equivalents. We did not find any significant correlations to symptoms or medication, inconsistent with the view that aberrant salience processing is linked to psychotic symptoms such as delusions and hallucinations (Kapur, 2003). The lack of correlations with symptoms may be due the limited range of clinical ratings as a result of our patients being chronic and clinically stable. Alternatively, the relatively small sample size could impact our power to detect effects in this type of analysis. These factors could similarly explain our lack of findings with regard to medication.

Another potential limitation of the current study is the apparent lack of an abnormal AB in patients, as both groups showed behavioral evidence of an AB. Although many previous studies report target detection deficits in schizophrenia, the results for the AB specifically are somewhat mixed. Some (Cheung et al., 2002; Li et al., 2002) but not all (Wynn et al., 2006; Mathis et al., 2011) behavioral studies have found evidence for an exaggerated AB effect in patients. When modified for use in an EEG paradigm (Mathis et al., 2012), schizophrenia patients exhibited a prolonged (i.e., longer lasting), rather than exaggerated (i.e., more pronounced) AB. Importantly, even without an exaggerated AB, patients showed a significantly reduced P300 ERP component indicating problems in attentional modulation. In summary, although an AB deficit is not always elicited behaviorally, patients with schizophrenia appear to exhibit neural abnormalities during complex visual target detection tasks such as the dual target RSVP, as we found in the current study.

This study complements and extends prior literature on the neural correlates of complex visual target detection deficits in schizophrenia. We found that individuals with schizophrenia showed abnormally increased deactivation of the ventral network during single target detection. In response to increased attention demands of dual target processing both patients and controls showed comparable activation in the dorsal and ventral networks. When comparing dual- to single-target processing, patients showed overactivation of the dorsal network along with failure to deactivate the ventral network, which could be driven by group differences in neural activation during the single target task. Patients have previously been shown to have deficits on single target detection tasks (e.g., oddball tasks), but it was unclear whether the difficulty of these tasks was sufficient to fully characterize the neural deficits associated with target detection in schizophrenia. Our findings suggest that increasing the difficulty of the target detection task by adding an additional target does not appear to further exacerbate patients’ deficits seen in less demanding single target tasks.

## Conclusion

The observed abnormal activation patterns during target detection may reflect inefficient and faulty suppression of salience-oriented processing regions, resulting in increased sensitivity to task stimuli and difficulty distinguishing targets from non-targets during attentional task demands. Along these lines, aberrant salience processing is suggested to be related to a hyperdopaminergic system (Kapur, 2003; van Os, 2009), one of the main neurotransmitters implicated in schizophrenia (Lisman et al., 2008). Further studies can assess the possible link between aberrant ventral network and dopaminergic systems in schizophrenia. Considering the close association between attentional abnormalities and poor functional outcome in schizophrenia, the abnormal neural activation pattern observed in this study could serve as a potential biomarker for treatments designed to improve target detection in individuals with this disorder.

## Author Contributions

AJ took the lead on data analysis, interpretation, and manuscript writing, including management of edits and revisions based on co-author feedback. MC, SE, DG, KN, and MG formulated the study concept and design, managed data collection, assisted in interpretation of data, and assisted with manuscript editing. MG, JL, JW, and ER assisted with data analysis planning, data interpretation, manuscript drafting, and editing.

## Conflict of Interest Statement

MG has been a consultant to AbbVie, DSP, Forum, and Roche, and he is on the scientific advisory board of Mnemosyne. He has received research funds from Amgen. KN has received unrelated research support from Janssen Scientific Affairs, Genentech, and Brain Plasticity, Inc., and has consulted to Genentech, Otsuka, Janssen, and Brain Plasticity, Inc. The rest of the authors report no biomedical financial interests or potential conflicts of interest.
