# Supplementary material for: Abnormal Ventral and Dorsal Attention Network Activity during Single and Dual Target Detection in Schizophrenia
Source: Front Psychol. 2016 Mar 8;7:323. doi: 10.3389/fpsyg.2016.00323 (PMC4781842; doi:10.3389/fpsyg.2016.00323)
Supplement: Supplementary file 1 [file Table_1.DOCX]

Supplementary Material

Abnormal Ventral and Dorsal Attention Network Activity During

**Single and Dual Target Detection in Schizophrenia**

**Amy M. Jimenez*, Junghee Lee, Jonathan K. Wynn, Mark S. Cohen, Stephen A. Engel, David C. Glahn, Keith H. Nuechterlein, Eric A. Reavis, Michael F. Green**

*** Correspondence:** Amy M. Jimenez: amjimenez@ucla.edu

## Supplementary Tables

**Supplementary Table 1.** Results of repeated-measures ANOVA on beta values from ventral and dorsal attention network ROIs during the single and dual target detection tasks.

| **Task** | | | **Group** | **ROI** | | **Group x ROI** |
| --- | --- | --- | --- | --- | --- | --- |
| ST Task, ventral network ROIs | | | F = 0.72 | F = 1.91 | | F = 3.05* |
| ST Task, dorsal network ROIs | | | F = 0.78 | F = 1.23 | | F = 0.87 |
| DT Task, all lags, ventral network ROIs | | | F = 0.02 | F = 5.82** | | F = 0.39 |
| DT Task, all lags, dorsal network ROIs | | | F = 0.16 | F = 1.01 | | F = 1.16 |
| DT Task, all lags > ST Task,  ventral network ROIs | | | F = 0.27 | F = 2.78^†^ | | F = 1.24 |
| DT Task, all lags > ST Task,  dorsal network ROIs | | | F = 0.27 | F = 2.26 | | F = 0.72 |
|  | | | | | | |
| DT Task, by lag | | | | | | |
| **ROI** | **Group** | **Lag** | | | **Group x Lag** | |
| ACC | F = 0.06 | F = 0.81 | | | F = 0.46 | |
| AI | F = 0.16 | F = 0.43 | | | F = 1.13 | |
| TPJ | F = 0.16 | F = 1.69 | | | F = 0.80 | |
| LFC | F = 0.02 | F = 0.35 | | | F = 0.10 | |
| aIPC | F = 0.01 | F = 0.50 | | | F = 0.62 | |
| pIPC | F = 0.79 | F = 0.65 | | | F = 0.76 | |
|  | | | | | | |
| DT Task > ST Task, by lag | | | | | | |
| **ROI** | **Group** | **Lag** | | | **Group x Lag** | |
| ACC | F = 1.21 | F = 0.55 | | | F = 0.17 | |
| AI | F = 0.04 | F = 0.61 | | | F = 2.50 | |
| TPJ | F = 6.42* | F = 1.36 | | | F = 0.38 | |
| LFC | F = 1.48 | F = 0.35 | | | F = 0.22 | |
| aIPC | F = 1.13 | F = 0.02 | | | F = 0.21 | |
| pIPC | F = 4.20* | F = 0.32 | | | F = 0.97 | |
|  | | | | | | |
|  | | | **Group** | **ROI** | | **Group x ROI** |
| DT Task, Lag 3 > Lag 1  ventral network ROIs | | | F = 0.43 | F = 1.74 | | F = 0.20 |
| DT Task, Lag 3 > Lag 1,  dorsal network ROIs | | | F = 0.32 | F = 2.09 | | F = 2.34 |
| DT Task, Lag 3 > Lag 7  ventral network ROIs | | | F = 2.92 | F = 1.31 | | F = 0.15 |
| DT Task, Lag 3 > Lag 7,  dorsal network ROIs | | | F = 0.04 | F = 0.21 | | F = 1.79 |

* *p* < .05; ***p* < .01; † *p* = .07. Abbreviations: ROI, region-of-interest; ST, single target; DT, dual target; ACC, anterior cingulate cortex; AI, anterior insula; TPJ, temporo-parietal junction; LFC, lateral frontal cortex; aIPC, anterior intraparietal cortex; pIPC, posterior intraparietal cortex.
